# Developmental hearing loss impairs signal detection in noise: putative central mechanisms

**DOI:** 10.3389/fnsys.2014.00162

**Published:** 2014-09-09

**Authors:** Jennifer D. Gay, Sergiy V. Voytenko, Alexander V. Galazyuk, Merri J. Rosen

**Affiliations:** ^1^Department of Anatomy and Neurobiology, Northeast Ohio Medical UniversityRootstown, OH, USA; ^2^Biomedical Sciences Program, Kent State UniversityKent, OH, USA

**Keywords:** conductive hearing loss, masking, noise, signal detection, auditory cortex, intracellular, electrophysiology, gerbil

## Abstract

Listeners with hearing loss have difficulty processing sounds in noisy environments. This is most noticeable for speech perception, but is reflected in a basic auditory processing task: detecting a tonal signal in a noise background, i.e., simultaneous masking. It is unresolved whether the mechanisms underlying simultaneous masking arise from the auditory periphery or from the central auditory system. Poor detection in listeners with sensorineural hearing loss (SNHL) is attributed to cochlear hair cell damage. However, hearing loss alters neural processing in the central auditory system. Additionally, both psychophysical and neurophysiological data from normally hearing and impaired listeners suggest that there are additional contributions to simultaneous masking that arise centrally. With SNHL, it is difficult to separate peripheral from central contributions to signal detection deficits. We have thus excluded peripheral contributions by using an animal model of early conductive hearing loss (CHL) that provides auditory deprivation but does not induce cochlear damage. When tested as adults, animals raised with CHL had increased thresholds for detecting tones in simultaneous noise. Furthermore, intracellular *in vivo* recordings in control animals revealed a cortical correlate of simultaneous masking: local cortical processing reduced tone-evoked responses in the presence of noise. This raises the possibility that altered cortical responses which occur with early CHL can influence even simple signal detection in noise.

## Introduction

Listeners with hearing loss often struggle to understand speech in noisy environments. This difficulty is reflected in increased thresholds for detecting a simple signal in noise, i.e., simultaneous masking. Sensorineural hearing loss (SNHL) affects both the peripheral (cochlear) and central auditory system (cochlear nucleus and above), making it challenging to determine the mechanisms underlying impaired signal detection. We thus use a model of conductive hearing loss (CHL) that leaves the cochlea intact, allowing us to determine whether and how central auditory changes induced by hearing loss affect signal processing in noise.

CHL has effects on the central auditory system. In children, chronic middle ear infections (otitis media) produce a fluctuating CHL that can overlap with critical periods of neural development. Auditory deprivation during these periods alters intrinsic cellular and synaptic properties throughout the central auditory system (Vale and Sanes, 2000, 2002; Leao et al., 2004; Youssoufian et al., 2005; Leão et al., 2006). Developmental CHL is correlated with persistent perceptual problems that are presumably linked to changes in the central auditory system (Whitton and Polley, 2011). Support for this idea arises from animal developmental studies (Knudsen et al., 1984; King et al., 2000; Popescu and Polley, 2010). For example, binaural CHL leads to increased perceptual detection thresholds for slow amplitude modulations, and these behavioral deficits match the magnitude of neural shifts in auditory cortex (ACx; Rosen et al., 2012).

Acoustically demanding conditions, such as noisy environments, are particularly challenging for children who experience CHL. In multiple studies, children with a history of CHL have greater difficulty correctly identifying words or understanding speech in background noise than controls, requiring higher signal-to-noise ratios (SNRs) to attain equivalent performance (Gravel and Wallace, 1992; Schilder et al., 1994; Hall et al., 2003; Eapen et al., 2008; Hsieh et al., 2009; Keogh et al., 2010). These speech processing difficulties are likely due to changes in the central auditory system that arise from auditory deprivation (Sanes and Bao, 2009).

Detection of simple signals in noise should also be susceptible to CHL-induced central auditory system changes. Auditory percepts that reach mature performance levels gradually are susceptible to central changes that can arise due to hearing loss-induced deprivation (Moore, 2002). In particular, for the detection of brief signals in noise (simultaneous masking), thresholds do not reach adult levels until 10 years of age or later in humans (Hartley et al., 2000; Huyck, personal communication).

Neural elements that are affected by early hearing loss may contribute to these deficits. In auditory cortex, many hearing loss-induced effects are considered to involve modifications in local inhibitory networks (Calford et al., 1993; Kral et al., 2000; Chang et al., 2005; Razak et al., 2008; Takesian et al., 2010, 2012). Importantly, at least some of these changes persist into adulthood (Takesian et al., 2012). Thus, cortical neural changes during development as a result of hearing loss may contribute to signal detection problems that persist into maturity.

Deficits in simultaneous masking are also seen in SNHL listeners, but are usually attributed to peripheral mechanisms. In SNHL listeners, elevated simultaneous masking thresholds are ascribed to broadened filters and abnormal intensity perception that arise from cochlear damage, particularly of outer hair cells (Moore and Glasberg, 1986; Glasberg et al., 1987; Florentine, 1992; Kidd et al., 2002; Oxenham and Bacon, 2003). In normal listeners, psychophysical models attribute signal-in-noise detection thresholds to processing within the cochlea (Dai et al., 1991; Schlauch and Hafter, 1991; Moore, 2012). However, both psychophysical and neurophysiological data indicate that the central auditory system contributes to simultaneous masking performance.

Several psychophysical phenomena and auditory cortical responses implicate the central auditory system in simultaneous masking. One is overshoot, when a signal presented in the middle of a noise masker is more detectable than a signal presented at or near the noise onset (Elliott, 1965; Zwicker, 1965). Peripheral mechanisms cannot explain this phenomena (Smith and Zwislocki, 1975; Moore et al., 1987; Bacon and Smith, 1991), but neurons in primary ACx have response properties consistent with overshoot: signals presented at a delay relative to a background sound elicited more action potentials than those presented close to background sound onset, and this was directly attributable to inhibition from local cortical circuits (Volkov and Galazyuk, 1992). Another compelling indicator of central involvement is that a subject’s expectation influences performance. In the presence of a continuous noise, detection of a tone can drop nearly to chance when the tone occurs at an unexpected frequency or duration (Scharf et al., 1987; Dai et al., 1991; Schlauch and Hafter, 1991; Wright and Dai, 1994). This susceptibility to stimulus variability has no peripheral correlate and implicates higher processing elements such as sensory memory and attention. A central correlate may exist in neurons from auditory cortical areas, which modulate their discharge rates in response to sound elements that deviate from expected values (Ulanovsky et al., 2003; Gill et al., 2008; Buran et al., 2014a).

Although it is difficult to pinpoint mechanisms underlying perceptual deficits, CHL, which does not result in hair cell damage, can disambiguate the contributions of peripheral and central elements to perception (Tucci et al., 1999; Lee et al., 2013). Early CHL induces central synaptic and cellular changes across the auditory neuraxis (Webster, 1983; Stuermer and Scheich, 2000; Tucci et al., 2001; Sumner et al., 2005; Xu et al., 2007; Takesian et al., 2010, 2012). Thus perceptual deficits resulting from CHL are presumably due to central contributions. Here we use an animal model of early CHL to demonstrate the effects of hearing loss on basic auditory perception, and to examine putative neural correlates in the central auditory system. Mongolian gerbils underwent surgery prior to the onset of hearing to induce a permanent moderate CHL. Animals with this permanent loss were then tested in adulthood compared with normal-hearing controls. Performance on an operant conditioning task demonstrated that early CHL impaired the perception of tones in simultaneous noise maskers. Then in normal-hearing animals, we used intracellular recordings to reveal a cortical correlate of simultaneous masking: local cortical processing reduced tone-evoked responses in the presence of noise. This raises the possibility that altered cortical responses affect simultaneous masking thresholds in animals with hearing loss.

## Methods

### Animals

All procedures relating to the maintenance and use of animals were approved by the Institutional Animal Care and Use Committee at the Northeast Ohio Medical University. Adult Mongolian gerbils (*Meriones unguiculatus*) ranging between postnatal (P) day 54–125 were tested in one of two procedures. Cortical responses to signals in noise were measured with intracellular recordings from a group of control (CTR) animals (*n* = 7). Perceptual detection thresholds were obtained from separate groups using an operant conditioning procedure. Control animals (*n* = 8) received normal auditory experience during development and were compared to animals with developmental CHL (*n* = 10). All animals were weaned at P30 and housed with litter mates in a 12 h light/12 h dark cycle. Groups were comprised of animals from multiple litters and included both males and females.

### Conductive hearing loss induction and measurement

#### Malleus removal

Bilateral CHL was induced at P10–11 prior to the onset of hearing by tympanic membrane puncture and malleus extirpation (Tucci et al., 1999). Pups were anesthetized with methoxyflurane (Metofane, Ivesco Holdings) and the malleus was removed bilaterally through perforations in each tympanic membrane. At the conclusion of the study, hearing thresholds were measured via auditory brainstem responses (ABRs) from all animals. In addition, CHL animals were sacrificed and both ears examined to confirm malleus removal and to verify that the cochlea was not damaged by visual inspection of the bony labyrinth.

#### Auditory brainstem response (ABR)

After behavioral testing, ABRs from a subset of animals were measured to assess neural hearing thresholds. Tucker Davis Technologies (TDT) software and hardware were used to generate and present sounds (SigGen, BioSigRZ, RZ6), and to digitize and record neural responses (RZ6, RA4PA). Animals were anesthetized with ketamine and chloral hydrate and presented with auditory stimuli from a free-field calibrated speaker (TDT MF-1) positioned 6 cm in front of the animal. Responses were measured using stainless steel needle electrodes inserted subdermally at the dorsal midline between the eyes (non inverting), posterior to each pinna (inverting), and base of the tail (common ground). Auditory stimuli were 3 ms pure tones with 1 ms rise/fall times, repeated at 15/s. Sound level was adjusted in 5 dB steps to obtain a threshold response (i.e., a visually detectable N1 potential).

### Behavioral training and testing

#### Experimental environment

Gerbils were placed in a custom built test cage in a double-walled room (ETS Lindgren Acoustic Systems) lined with echo-attenuating material, and were observed via closed circuit monitor. The test cage contained a stainless steel drinking spout and metal floor plate. Gerbil contact with both plate and spout completed a circuit that initiated water delivery via a syringe pump (New Era). A personal computer connected to a digital I/O interface TDT measured animal contact and controlled the timing of acoustic stimuli, water delivery (0.2–0.25 ml/min), and a small aversive current delivered at the end of warning trials. Auditory stimuli were generated by the TDT system and delivered via a calibrated custom speaker (Madisound) centered ~ 60 cm in front of the lick spout. Sound level at the test cage was measured with a spectrum analyzer (Bruel and Kjaer 2690-OF2) via a 1/4 inch free-field condenser microphone (Bruel and Kjaer 4939) positioned at the location of the animal head when in contact with the spout. Noise levels are in dB SPL, converted from RMS measurements.

#### Auditory stimuli and operant conditioning task

**Figure 1:**
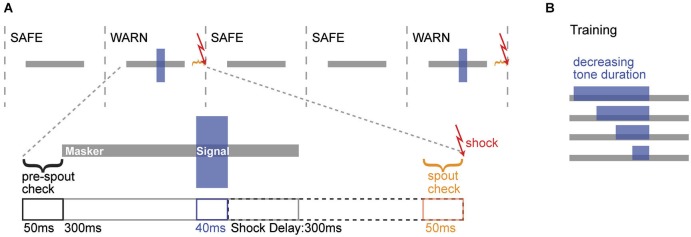
**Operant conditioning schematic**. **(A)** *Top*: Trial structure for the simultaneous masking task, where 300 ms noise maskers were presented either with (WARN trial) or without (SAFE trial) overlapping tonal signals. Warn trials were followed by an aversive shock. *Bottom*: The timeline of a single warn trial is illustrated, with the masker (*gray*) and signal (*blue*) just above. For the trial to be initiated, the animal needed to maintain contact with the spout for >75% of the pre-spout check period. WARN trials contained a 300 ms masker and a 40 ms signal. A mild shock was delivered 300 ms after the offset of the signal. During the 50 ms prior to the shock, a spout check determined whether the animal was correctly off the spout (Hit) or incorrectly on the spout (Miss). For SAFE trials (not illustrated), neither the signal nor shock were presented, but the spout check determined whether the animal was correctly on the spout (Correct Rejection) or incorrectly off the spout (False Alarm). **(B)** During training, the duration of the signal was progressively decreased as animals reached criterion performance at each duration. The shock always occurred 300 ms after signal offset, regardless of signal duration.

During training and testing, animals heard continuous repeated bursts of a 300 ms noise masker (30% BW noise centered linearly at 4 kHz) with 700 ms inter-burst intervals. Within this repeated background were intermittent SAFE and WARN trials (Figure 1A). SAFE trials contained only the noise masker, while WARN trials contained the masker and signal (4 kHz pure tone, 40 ms duration, 2 ms rise/fall; Figure 1A). Contact with the spout was monitored immediately prior to each trial and the trial only proceeded if the animal was in contact with the spout for 75% of the 50 ms pre-check window. The warning stimulus was followed by an aversive unconditioned stimulus (300 ms electrical current via the lick spout) delivered 300 ms after the end of the 40 ms signal. To determine if the animal detected the signal, contact with the spout was monitored during the 50 ms prior to delivery of the shock. Contact for ≤75% of this window was scored as a hit. For the same window during SAFE trials a contact time of ≤75% was scored as a false alarm (FA). WARN trials always occurred at the end of a block of 2–4 SAFE trials, randomized to avoid temporal conditioning.

#### Sound levels

Control and CHL animals were tested at approximately equivalent sensation levels based on previous measures of hearing loss induced by malleus removal. This procedure typically produces an attenuation of ~45 dB as assessed by ABR (Tucci et al., 1999; Rosen et al., 2012), and of ~30 dB at 4 kHz as assessed by behavioral testing (Buran et al., 2014b; see Section Results for more detail). Sound levels were presented 35 dB louder for CHLs (85 dB SPL masker) than CTRs (50 dB SPL masker). The signal levels began at 23 dB SPL above the masker level and were reduced to determine detection threshold. We determined that the stimulus was not distorted at the loudest levels presented.

#### Procedural training

Behavioral training and testing involved a conditioned avoidance procedure (Heffner and Heffner, 1995; Kelly et al., 2006). Animals were water deprived for 48 h prior to training and remained on controlled water access for the duration of the training and testing. Animals were introduced to the behavioral cage and trained to initiate water delivery via contact with the metal spout, during repeated presentations of gated noise maskers (Figure 1A). Animals learned to withdraw from the spout when an acoustic cue (tonal signal) was presented within the noise, in order to avoid a low AC current (0.25–2.5 mA, 300 ms, Coulbourn) delivered through the lick spout. Since animals display large between-subject variability in pain sensitivity (Mogil, 1999; Wasner and Brock, 2008; Nielsen et al., 2009), the shock level was adjusted continuously for each animal to produce reliable withdrawal from the spout without dissuading the animal from returning to the spout. For initial training, a long (200 ms) signal was presented and shortened in 20–30 ms steps until animals reliably detected the target duration of 40 ms (Figure 1B). To establish criterion performance at the target duration, warning trials were presented until performance reached 70% correct over 10 consecutive trials.

#### Perceptual testing

Once animals reached criterion on the conditioned avoidance procedure, they were tested with decreasing signal levels using the method of constant limits: five signal levels separated by 3 dB presented in decreasing order. Each day of testing used a range of sound levels that bracketed the previous day’s threshold (Sarro and Sanes, 2010). Animals were tested for 4–5 days with increasing difficulty in order to determine thresholds for detecting the signal in noise (signal-to-noise ratio, SNR, quantified as the signal in dB minus the noise in dB). Pilot data indicated that this duration of testing produced reliable performance, while longer testing resulted in poorer performance, as the difficulty of the task induced animals to adopt strategies that resulted in increased thresholds (e.g., high FA rates or long lapses of attention). SNRs for the best day along with the best 3 days of performance were taken as perceptual thresholds.

#### Data analysis

A performance value, d′ = *z*(hits)−*z*(false alarms), was obtained for z-scores that corresponded to right-tail *p*-values (Swets, 1973; Yanz, 1984), and was calculated for each signal level. Thresholds were defined as the signal level at which performance reached d′ = 1; only sessions in which animals performed on a minimum of 25 WARN trials were included in the analysis. Psychometric functions of d′ across signal level were constructed for each day of testing. Performance during the best 3 days of testing served as the assessment of practiced detection thresholds. Performance across groups was compared with two-sided Wilcoxon rank sum tests for unpaired data. All values are given as mean ± standard error (SEM).

#### Inclusion criteria

All animals that performed behavioral testing were included in the analysis. In order to compare the effects of treatment group, no animals were excluded for poor performance, as is common in behavioral studies examining best performance capability. Animals that did not reach criteria after 10 days of training with the long-duration signal were excluded. This removed three controls and six CHL animals from the study.

### Intracellular recordings

#### Surgical preparation

Mongolian gerbils (*n* = 7) were anesthetized through isoflurane inhalation (1.5–2.5% in oxygen) and held secure in a stereotaxic apparatus. A headpost was cemented to the skull using dental acrylic, and a small craniotomy was made over left auditory cortex, leaving the dura intact. Following this brief surgical procedure, animals were anesthetized with ketamine (30 mg/kg) and chloral hydrate (350 mg/kg) in preparation for recordings, and were maintained as necessary with supplemental doses.

#### Acoustic stimuli

Stimuli were generated using TDT software (SigGen) and hardware (RP 2.1) and delivered via a calibrated custom built speaker. The signal was a 40 ms FM downsweep with a 10 kHz bandwidth, extending through a range chosen to encompass the cell’s best frequency (BF). This range was based on an initial extracellular recording that determined the general BF of cells within the region. The masker was a 200 ms broadband noise, with onset 100 ms before the FM signal. Signal and masker were presented at equal amplitudes.

#### Electrophysiology

Intracellular recordings were made using techniques described previously (Voytenko and Galazyuk, 2008). Animals were placed inside a single-walled acoustic chamber (Industrial Acoustics), and positioned on an air table 7″ from a freefield speaker. Sharp microelectrodes were pulled from 1.2-mm-diameter quartz glass (Sutter Instruments) on a Flaming-Brown micropipette puller and filled with 3M potassium acetate. Impedance ranged between 40 and 90 MΩ. After placement on the dorsal surface of the brain, the exposure was filled with 4% agar and the electrode was advanced in 3-μm steps using a precision microdrive (Kopf, Model 660). Intracellular responses were amplified (Cygnus Technologies NeuroData IR183A), monitored with Pulse software (v. 8.65) and digitized using a data acquisition system (Heka model EPC-10) at a sampling rate of 100 kHz.

#### Analysis

Responses to masked and unmasked signals were compared within cells. 10–20 repetitions were presented for each stimulus, and mean traces calculated from membrane potentials (V_m_) after truncating spikes. Hyperpolarization within a given time window was measured from mean traces as the area under the curve in relation to baseline V_m_. For each cell, the time window in which a specific response occurred (either a hyperpolarization or rebound action potentials) was determined from the mean V_m_ trace elicited by the signal alone. This time window was used to measure response magnitudes with and without a masker present; these were compared with two-sided Wilcoxon signed rank tests for paired data. All values are given as mean ± SEM.

## Results

### Developmental conductive hearing loss degrades signal detection in noise

**Figure 2:**
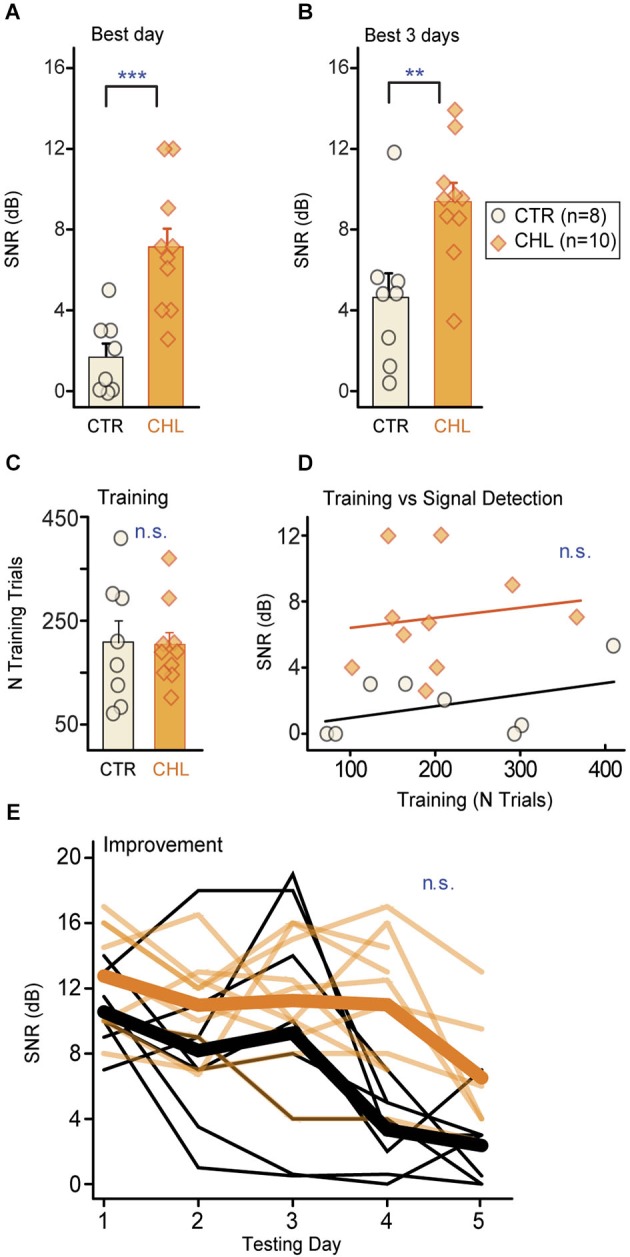
**Early CHL increases behavioral detection thresholds for simultaneously masked signals**. Detection thresholds (quantified as SNR; see Section Methods) were significantly higher for conductive hearing loss (CHL; *shaded orange*) compared with control (CTR; *cream-filled* *black*) animals, as measured by **(A)** the best performance day and **(B)** the mean across the best 3 performance days. Thresholds from individual animals are depicted as *circles* or *diamonds* atop each bar. **(C)** Groups required similar numbers of training trials to reach criterion performance. **(D)** The amount of training did not predict final detection thresholds. *Lines* show non-significant linear fits. **(E)** Detection thresholds across testing days indicate gradual improvement which did not differ across groups. *Thin lines* are thresholds from individual animals, and *thick lines* are means. Abbreviations: CTR: controls, CHL: conductive hearing loss, n.s.: not significant, **: *p* < 0.01, ***: *p* = 0.001.

Behavioral thresholds for signal detection in noise were obtained from two groups of adult gerbils: animals reared with CHL prior to hearing onset, and age-matched controls (CTR). Animals were trained to detect a brief 4 kHz signal embedded in a longer noise masker (30% bandwidth centered around the signal frequency). Once animals were reliably performing the task, they received several days of procedural testing to determine threshold (defined as the quietest tone level that could be reliably detected in the presence of the masker, reported here as SNR in dB). Thresholds were compared across groups for each animal’s best day (Figure 2A) and mean across the best 3 days (Figure 2B). CTR animals displayed significantly lower detection thresholds compared to CHLs (Best day: *p* < 0.001; Best 3 days: *p* = 0.01, Wilcoxon rank-sum). Differences in thresholds were not due to differences in training or performance on the task. Groups received a comparable number of procedural training trials (Figure 2C: *p* = 0.97, Wilcoxon rank sum) and learned the task at a similar rate (days of training: CTR 5.6 ± 0.9 vs. CHL 5.8 ± 0.6, *p* = 0.69, Wilcoxon rank-sum). Furthermore, the amount of procedural training on the task was not correlated to threshold for either group (Figure 2D: CTR: *R*^2^ = 0.192, *p* = 0.28, CHL: *R*^2^ = 0.021, *p* = 0.69).

One might expect that early hearing loss would affect learning or improvement on perceptual tasks. For this simple simultaneous masking task that was not the case. In addition to equivalent time-courses of training, both groups improved but did so equivalently across testing days (Figure 2E; First day minus last day threshold (dB SNR): CTR 7.8 ± 2.7 vs. CHL 4.7 ± 4.6, *p* = 0.15, Wilcoxon rank-sum). Furthermore, the rate of learning did not differ between groups, as animals reached best performance over an equivalent number of testing days (number of testing days to reach threshold (dB SNR): CTR 4.6 ± 0.2 vs. CHL 3.7 ± 0.4, *p* = 0.19, Wilcoxon rank-sum).

### Performance variables do not account for the effect of early hearing loss

**Figure 3:**
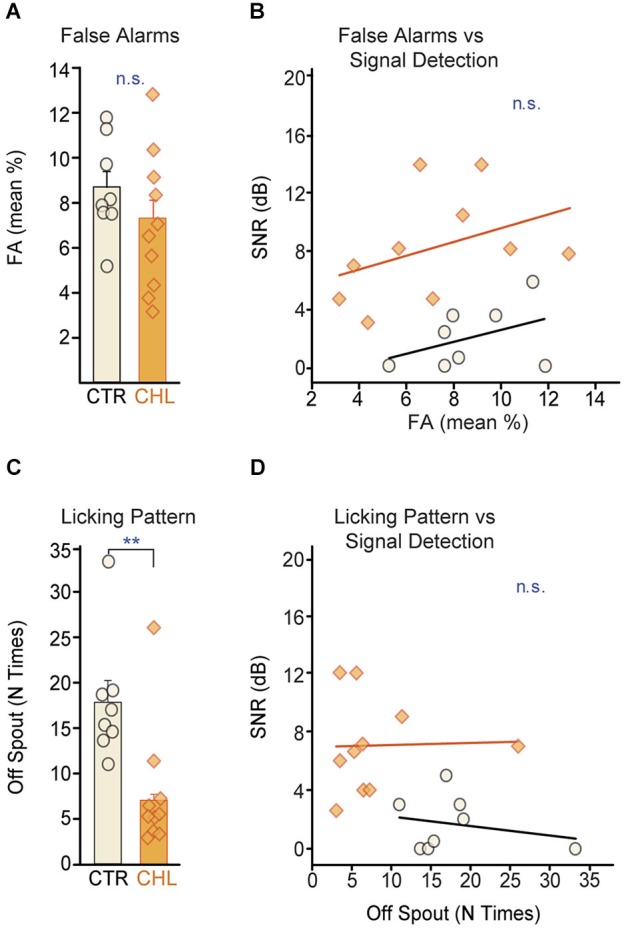
**Performance strategies do not account for differences in behavioral detection thresholds**. **(A)** False alarm (FA) rates, a measure of attention, did not differ across CTR (*open black*) and CHL (*shaded orange*) animals. FA rates from individual animals are depicted as *circles* or *diamonds* atop each bar. **(B)** FA rates did not correlate with behavioral detection thresholds for either group (*lines* show non-significant linear fits). **(C)** CTR animals adopted a less consistent licking strategy than CHL animals (quantified as the number of breaks with the water spout between WARNs: N times off spout). **(D)** Despite a difference in licking stragegy, the number of breaks did not correlate with final detection thresholds for either group (*lines* show non-significant linear fits). Abbreviations as in Figure 2.

Higher thresholds for the CHL animals did not appear to be due to poorer attention to the task. Poor attention can be indicated by high FA rates, measured here as withdrawal from the spout during SAFE trials (which minimizes the chance of receiving a shock during poor attention to the signal). CTR and CHL animals did not differ in their FA rates (Figure 3A: *p* = 0.26, Wilcoxon rank-sum) and this measure of attention did not predict threshold for either group (Figure 3B: CTR: *R*^2^ = 0.172, *p* = 0.31, CHL: *R*^2^ = 0.159, *p* = 0.25).

Attention can also be indicated by licking consistency. During testing, animals ideally maintain constant contact with the spout, and withdraw only upon detecting a signal. Another strategy is to drink continuously but make poor contact with the spout, with continuous micro-withdrawals to minimize the magnitude of shock received. This hesitant contact can be an indicator of poor attention or performance anxiety and can be quantified as the number of times an animal breaks contact with the spout between WARNs. CTR animals displayed significantly more breaks in contact between WARNs compared to CHLs (Figure 3C: *p* < 0.01, Wilcoxon rank-sum) indicating group differences in overall performance strategy. However, this was independent of signal detection thresholds (Figure 3D: CTR: *R*^2^ = 0.06, *p* = 0.57, CHL: *R*^2^ = 0.001, *p* = 0.93). Notably, this performance strategy would be likely to increase the false alarm rate in CTR animals, thus increasing their thresholds as calculated by the d′ measure. Despite this, CTR thresholds were significantly lower than those of CHLs, suggesting that, if anything, the threshold differences measured here may underestimate perceptual differences across groups.

**Figure 4:**
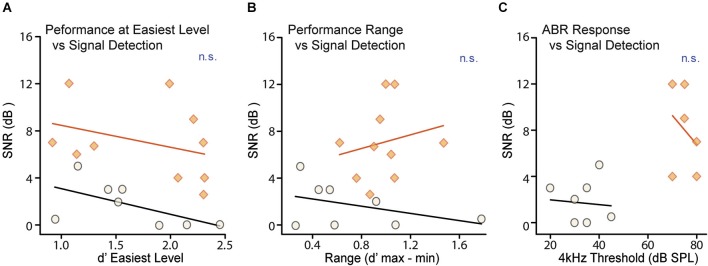
**Neither task proficiency nor hearing ability account for differences in detection thresholds**. Detection thresholds were not correlated with either **(A)** d′ performance for the easiest signal levels or **(B)** the variability of d′ performance for the easiest signal level across testing days, for either CTR (*shaded orange*) or CHL (*open black*) groups. Thresholds from individual animals are depicted as *circles* or *diamonds*, and *lines* are non-significant linear fits. **(C)** Neural ABR thresholds measured across frequency were predictably increased in CHL animals by ~40 dB. There was no correlation in either CTRs or CHLs between neural hearing thresholds and signal detection thresholds. Abbreviations as in Figure 2.

Task proficiency and performance consistency can also affect behavioral thresholds. As an indicator of task proficiency, we measured performance at the easiest level on the best testing day (Figure 4A). As an indicator of performance consistency we measured the range of d′ scores for the easiest level presented across days; a wider range indicates increased variability in performance (Figure 4B). Neither the d′ score for the easiest level on the best performance day (CTR: *R*^2^ = 0.35, *p* = 0.12, CHL: *R*^2^ = 0.11, *p* = 0.34) nor the range of d′ scores at the easiest level (CTR: *R*^2^ = 0.17, *p =* 0.30, CHL: *R*^2^ = 0.42, *p* = 0.57) correlated with behavioral threshold for either group. Thus perceptual deficits in CHL animals are independent from their ability to perform the task.

### Sensation level alone does not account for the effect of early hearing loss

In order to attribute deficits in masked thresholds to central changes, it is essential to demonstrate that the attenuation provided by CHL is not sufficient to explain these deficits. It is possible that increased signal detection thresholds for CHL animals could be attributed to the stimuli not being presented at sensation levels equivalent to CTRs. To account for this, the two groups were tested with stimuli that differed by 35 dB (for both masker and signal). This level difference was based on behaviorally-measured level thresholds for separate groups of CHL animals in two tasks: AM detection (Rosen et al., 2012) and tone-detection (Buran et al., 2014b), which indicated 35 and 30 dB shifts respectively. We then tested to ensure that the amount of hearing loss at 4 kHz did not predict detection thresholds for our 4 kHz masked tones. Figure 4C shows that hearing levels for a 4 kHz tone, as measured by ABRs for each animal, did not correlate with behavioral thresholds in either CTRs (*R*^2^ = 0.01, *p* = 0.84) or CHLs (*R*^2^ = 0.10, *p* = 0.55). As there was no differential difficulty in hearing the signal, non-equivalent sensation levels could not account for the increased masked thresholds seen in CHL animals. That is, the characteristics of the perceptual deficit are not what would be predicted based solely on the amount of signal attenuation caused by the CHL.

### Auditory cortex contributes to reduced responses during simultaneous masking

Since the mechanisms by which central areas contribute to simultaneous masking are not resolved, intracellular responses to noise-masked signals were measured via sharp electrode recordings in auditory cortex. We focused on signal-evoked hyperpolarizations, as these are of cortical origin and reflect central processing (Somogyi et al., 1983; DeFelipe and Jones, 1985; Matsubara, 1988; Albus et al., 1991; Albus and Wahle, 1994; Tomioka et al., 2005; Higo et al., 2007). The spikes that occurred when hyperpolarizations returned to baseline were examined because they were likely to be generated by post-inhibitory rebound. Such spikes can be attributed to local cortical processes.

**Figure 5:**
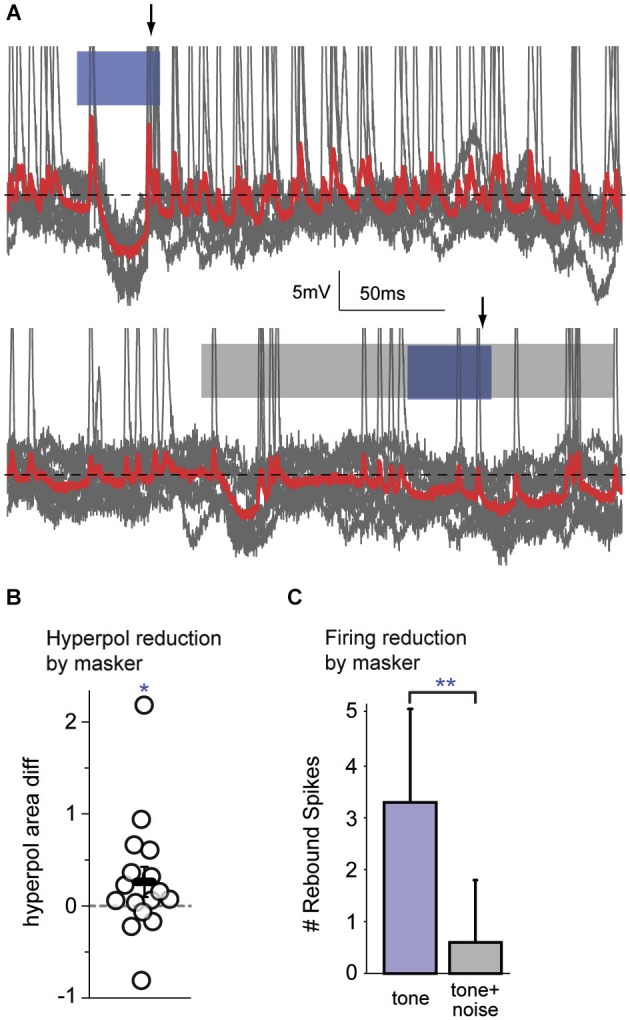
**Auditory cortex alters firing responses to simultaneous masked signals**. **(A)** An example of overlaid traces (*gray traces* with mean in* red*) from a cortical neuron in response to 10 presentations of a FM tone (*top; blue bar*) or a FM tone occurring during a broadband noise (*bottom; blue bar within gray bar)*. The traces show changes in voltage over time. *Top*: This neuron has an onset spiking response to the FM tone followed by a hyperpolarization, with rebound spikes (*arrow*) upon return to baseline (*black dotted line*). *Bottom*: When a simultaneous masker is presented, there is a reduced hyperpolarization and no clear rebound spikes (*arrow*) in response to the masked FM signal. **(B)** During masker presentation compared with tone alone, there was a significant reduction in the magnitude of signal-evoked hyperpolarizations. **(C)** When a simultaneous masker was present (*gray bar*), there were significantly fewer spikes immediately following hyperpolarizations, as compared with the tone presented alone (*blue bar*). Since hyperpolarizations arise within cortex and indicate central processing, a reduction in their magnitude, and a reduction of the number of post-inhibitory rebound spikes, are attributable to central mechanisms. **: *p* < 0.01, *: *p* < 0.05.

The subset of cells (*n* = 16) that exhibited hyperpolarization in response to the signal were examined. For each cell, the response to the signal alone was compared with the response to the masked signal. An example of overlaid traces in Figure 5A (top) shows a FM signal-evoked spiking onset response followed by a hyperpolarization, with rebound spikes upon return to baseline. The bottom trace shows a reduced hyperpolarization with no clear rebound spikes in response to the FM signal during a masker. For the sample of cells tested, the magnitude of the signal-evoked hyperpolarization (measured as the area under the curve relative to the membrane resting potential) was significantly reduced during presentation of the masker (Figure 5B; Wilcoxon signed rank, *p* = 0.049). Furthermore, those cells that spiked on rebound from the FM signal-evoked hyperpolarization fired significantly fewer rebound spikes during masker presentation (Figure 5C; Wilcoxon signed rank, *p* = 0.008). A reduced firing response to a masked signal is one measure of a neural correlate of perceptual masking. As this signal-evoked hyperpolarization must arise locally, from intrinsic cortical cellular mechanisms or local inhibitory circuitry, these data are evidence for a cortical contribution to simultaneous masking.

## Discussion

This study demonstrates that simultaneous masking involves the central auditory system and is impaired by early CHL. Developmental auditory deprivation induces anatomical and physiological changes in the central auditory system that persist into adulthood in animals (Harrison et al., 1993; Chang and Merzenich, 2003; Kral et al., 2005; Sanes and Bao, 2009). Perceptions that involve the central auditory system should be affected. We thus asked whether animals with early CHL had increased detection thresholds for simple signals in noise. When tested with a conditioned avoidance procedure, adult animals reared with CHL displayed significantly poorer detection thresholds than CTRs for a brief tone embedded in a simultaneous noise masker, despite equivalent training (Figure 2). These masked threshold increases could not be attributed to the amount of attenuation induced by the hearing loss. Furthermore, they were not due to task proficiency, strategy, or attention, as these did not differ across groups (Figures 3, 4). This type of developmental CHL maintains cochlear integrity and is known to alter intrinsic and synaptic properties in the central auditory system, particularly in ACx (Xu et al., 2007; Takesian et al., 2010, 2012). For such alterations to affect thresholds for simultaneous masking in deprived animals, neural areas central to the periphery should directly contribute to the responses to these signals, rather than merely inheriting responses from the auditory periphery. We therefore made intracellular *in vivo* recordings from ACx in control animals, and demonstrated a cortical contribution to simultaneous masking. This can be attributed to local sources, either intrinsic neural properties or local inhibitory synaptic inputs (Figure 5; Somogyi et al., 1983; DeFelipe and Jones, 1985; Matsubara, 1988; Albus et al., 1991; Albus and Wahle, 1994; Tomioka et al., 2005; Higo et al., 2007).

### Technical considerations of the developmental CHL model

The CHL induced here, via malleus removal, attenuates sound transmission to the inner ear but leaves the cochlea intact: ossicle removal does not alter hair cell counts on the basilar membrane, and bone conduction thresholds are normal (Tucci and Rubel, 1985). Here, visual postmortem analyses excluded the possibility of accidental cochlear damage. In SNHL listeners, reduced cochlear nonlinearities are often used to explain raised thresholds for simultaneous masked signals (Moore and Glasberg, 1986; Oxenham and Bacon, 2003), but this should not be the source of the raised thresholds seen here. The possibility exists that the permanent CHL induced compensatory changes at the periphery, perhaps via efferent alterations of the middle ear reflex (Munro and Blount, 2009). However, signal detection thresholds were not correlated with hearing thresholds at the same frequency (Figure 4C). Another possible peripheral contribution would be the induction of a hearing loss that was not consistent across the frequency range used in the behavioral masking task. Hearing loss with malleus removal is relatively flat across the frequency spectrum (Rosen et al., 2012), mimicking the flat CHL that occurs during otitis media in children (Kokko, 1974; Anteby et al., 1986; Hunter et al., 1994). Here, signals of 4 kHz were masked with a noise spanning 3.4–4.6 kHz, which approximates the gerbil critical band at that center frequency (~1 kHz; Kittel et al., 2002). Finally, it is worth noting that CHL induced via malleus removal is not the best mimic of otitis media, but is well-suited to examine central contributions to hearing loss deficits. Our CHL is permanent, whereas early otitis media typically produces a fluctuating CHL that resolves after childhood. Our model does not reproduce the effusion viscosity that occurs with otitis media, which alters sound transmission delays (Hartley and Moore, 2003). It therefore avoids introducing an element of altered peripheral processing that may affect temporal processing in listeners with otitis media.

### Cortical contributions to simultaneous masking

Our evidence in combination with previous work indicates that responses to simultaneous masked signals evolve from the periphery to central regions. In the periphery, simultaneous masking functions in auditory nerve fibers are based upon cochlear nonlinearities. For example, with a continuous broadband noise masker, auditory nerve fiber responses to tones shift in a manner consistent with two-tone suppression (the reduction of the response to one tone by simultaneous presentation of another tone) (Costalupes et al., 1984; Ruggero et al., 1992). This suppression is attributable to outer hair cell interactions with the basilar membrane (Robles and Ruggero, 2001). SNHL reduces those nonlinearities, which affects simultaneous masked thresholds (Oxenham and Bacon, 2003). In comparison, CHL, which does not alter cochlear nonlinearities, also affects simultaneous masked thresholds, and this is presumably due to central processes.

Our intracellular data reveal a cortical correlate of simultaneous masking. In neurons that exhibited signal-evoked hyperpolarization, the responses to FM tones showed a reduced hyperpolarization and fewer subsequent spikes in the presence of a noise masker (Figures 5B,C). The action potentials that immediately follow the hyperpolarization are likely to be rebound spikes, resulting from intrinsic cellular properties and thus of cortical origin. Cortical inhibition is known to arise locally, via intrinsic horizontal, intralaminar, and some long-range projections (Somogyi et al., 1983; DeFelipe and Jones, 1985; Matsubara, 1988; Albus et al., 1991; Albus and Wahle, 1994; Tomioka et al., 2005; Higo et al., 2007). As the hyperpolarizations seen here arise locally, our data are an example of the cortex modifying inherited subcortical information. The two possible sources of this hyperpolarization are either inhibition from local interneurons, or intrinsic cellular properties that generate afterhyperpolarizations. Intracellular *in vivo* recordings of ACx pyramidal neurons show responses similar to those seen here: signal-evoked depolarization often followed by hyperpolarization. The hyperpolarization has characteristics of a chloride-mediated IPSP that is likely mediated by GABA_A_ receptors (De Ribaupierre et al., 1972; Ojima and Murakami, 2002). Here, the hyperpolarization time courses were similar to those previously described. Therefore they are likely to arise from local inhibition rather than intrinsic hyperpolarizing currents activated by depolarization. If the hyperpolarization indeed arises from local cortical interneurons, the reduced hyperpolarization seen during masking may arise from reduced excitatory input onto those interneurons. Reduced neural responses are seen in subcortical regions during simultaneous masking, and may be a source of these excitatory feedforward inputs (Costalupes et al., 1984; Rees and Palmer, 1988; May and Sachs, 1992).

To our knowledge, the only other direct demonstration of inhibitory reduction of spiking during simultaneous masking in ACx is by Volkov and Galazyuk (1992), where a background sound evoked a local inhibitory current that reduced the response to an overlaid signal. However, they did not directly compare unmasked and masked conditions and only tested binaural interactions. Previous neurophysiological analyses of ACx and inferior colliculus have provided some evidence for central contributions to simultaneous masking involving local inhibition. In the inferior colliculus, tone-evoked responses were shifted during simultaneous noise in a manner consistent with local inhibition, providing responses that differed from auditory nerve fibers (Rees and Palmer, 1988). In primary ACx, monotonic cells (those whose discharge rate increases with sound level) responded like auditory nerve fibers, dominated by whichever sound (signal or masker) elicited a stronger response alone. In contrast, nonmonotonic cells (those with more complex level-sensitivity) were suppressed by a noise masker, suggestive of inhibitory influences (Phillips and Cynader, 1985; Phillips and Kelly, 1992). Consistent with this idea, nonmonotonic response profiles do not reflect inputs inherited from lower hierarchical areas. Instead, they arise from local excitatory and inhibitory interactions (Wu et al., 2006). Thus our result is expected: local cortical inhibition shapes responses to signals masked by simultaneous noise. We did not explicitly test for monotonicity, but would predict that the neurons analyzed here were nonmonotonic.

### Neural correlates of signal detection

A challenging element is connecting changes in neural discharge with altered auditory perception. The reduced spiking shown in this study indicates a reduced output to a masked signal and would be expected to increase signal detection thresholds. This idea is supported by recordings of cortical auditory evoked potentials (CAEPs) in humans, which show a correlate for the detection of masked signals in the P1/N1/P2 waveform. The amplitude of CAEP peaks is correlated with detectability of signals in noise (Martin et al., 1997). Since the CAEP primarily reflects synchronous activity arising from ACx (Näätänen and Picton, 1987; Eggermont, 2007), the reduction in ACx spiking seen here during the presence of a masker could manifest as reduced CAEP amplitude, and may thus contribute to poor perception in noise.

Cortical changes known to occur with hearing loss suggest a simple mechanism for impaired signal detection. Early CHL directly affects the central auditory system, generally reducing inhibition relative to excitation in order to maintain homeostasis (Sanes, 2013). For example, animals raised with early CHL have reduced IPSC amplitude, decreased firing rates in fast-spiking interneurons, and facilitating rather than the normal depressing short-term plasticity (Takesian et al., 2010, 2012). Inhibition is known to sharpen neural tuning curves at the level of cortex (Ojima, 2011). In normal listeners, inhibition would reduce the magnitude of noise-evoked responses. With reduced cortical inhibition seen from early hearing loss, the frequency components of a noise surrounding a neuron’s BF would *increase* the response magnitude to noise. At the same time, early CHL reduces the magnitude of ACx firing to tones (Rosen et al., 2012). Concurrently, these effects would reduce the SNR of tones presented in a noise masker. In this manner, changes to the cortical network may subserve the impaired simultaneous masked thresholds seen in this study. This prediction can be directly tested in our animal model. Future experiments that measure neural activity during perceptual detection tasks in CHL animals can connect changes in central auditory regions with perceptual impairments arising from developmental deprivation.

## Conflict of interest statement

The authors declare that the research was conducted in the absence of any commercial or financial relationships that could be construed as a potential conflict of interest.
